# Critically ill patients with 2009 H1N1 infection in an Indian ICU

**DOI:** 10.4103/0972-5229.68220

**Published:** 2010

**Authors:** J. Chacko, B. Gagan, E. Ashok, M. Radha, H. V. Hemanth

**Affiliations:** **From:** Multidisciplinary ICU, Manipal Hospital, Bangalore, India

**Keywords:** H1N1, influenza, virus, intensive care

## Abstract

**Background and Aims:**

The 2009 pandemic influenza A (H1N1) has taken its toll across most parts of India. We aimed to study its epidemiology, clinical characteristics and outcomes from an Indian multidisciplinary intensive care unit (ICU).

**Materials and Methods:**

All patients admitted to our ICU with a flu-like illness and who tested positive for the 2009 H1N1 by reverse -transcriptase polymerase- chain -reaction assay during a 3 month period were prospectively studied.

**Results:**

Thirty one patients were admitted to the ICU during the study period. Patients were in the younger age group with a median age of 35 years (IQR: 28.2-42.8). Obesity was the commonest risk factor. Twenty six patients (83.9%) required ventilator support; the median duration of ventilator support was 10 days (IQR: 4-22). Severe hypoxemia was the predominant feature in all patients. Circulatory failure requiring vasopressors occurred in 18 (58.1%) patients and acute kidney injury in 6 (3.2%) patients. Twenty six patients were alive at the end of 28 days; subsequently all except one were discharged. The median duration of hospital stay was 15 (IQR: 8-22.5) days. Increasing APACHE II scores were associated with an increased risk of death (Hazard Ratio: 1.1; CI: 1.08 -1.2; *P* = 0.04). Mean tidal volumes in non-survivors were significantly lower; this was related to poor lung compliance in this group.

**Conclusions:**

2009 H1N1 infection caused severe disease in relatively young patients without significant co-morbidities, characterized by severe hypoxemia and the requirement for prolonged mechanical ventilation. Extra-pulmonary organ failure included circulatory and renal failure.

## Introduction

The first reports of the 2009 H1N1 virus were confirmed in California by the Centre for Disease Control and Prevention (CDC) in April 2009.[1] According to the World Health Organization (WHO) update of 20^th^ November 2009, the virus has spread across more than 206 countries resulting in over 6,750 deaths.[2] In June 2009, the WHO raised the level of pandemic alert to phase 6, considering the sustained community level transmission of the virus across several regions.[3] According to the Directorate General of Health Services, Government of India, New Delhi update of 19^th^ November 2009, there have been 16,044 laboratory confirmed cases recorded in India, resulting in 537 deaths.[4] These numbers are likely to be an underestimate, as laboratory testing was restricted to hospital inpatients during the latter part of the outbreak and several cases in the community may have been unreported. The state of Karnataka recorded 119 deaths, which is the second highest for the country.[4] We received the first case of laboratory confirmed H1N1 at our hospital in August 2009. Since then, the number of new cases rose steadily, before touching a plateau by early October, followed by a decline. All critically ill adult patients were taken care of in our Multidisciplinary Intensive Care Unit (MICU). We report on the epidemiological and clinical characteristics and outcomes of the first 31 patients admitted to our MICU during this period.

## Materials and Methods

The MICU of our hospital is a 23 bedded, closed unit, staffed by full time intensivists and trainees in critical care medicine. We prospectively collected data on all confirmed H1N1 cases admitted during a three month period from August to October 2009. All patients presenting to our Emergency Department (ED) with an influenza-like illness suspicious of H1N1 infection had nasopharyngeal and throat swabs taken and preserved in a virus transportation medium before being analyzed at the regional testing centre at the National Institute of Mental Health and Neurological Sciences (NIMHANS), Bangalore. Testing was done by Reverse- Transcriptase Polymerase -Chain -Reaction (RT-PCR) assay as recommended by the CDC.[5] Patients with suspected H1N1 infection were transferred to the MICU based on the judgment of the attending physician in the ED at initial presentation or later on upon deterioration in the wards. All patients who presented with a respiratory illness requiring ICU care and tested positive for pandemic H1N1 infection by RT-PCR were included in the study.

Patients admitted to the MICU were cared for in one of three individual cubicles as much as possible, but were accommodated in an earmarked, open area, in case of spill over. Treatment included oseltamivir along with ceftriaxone and azithromycin as empirical antibacterial cover. Decisions regarding mechanical ventilation, vasopressor support or renal replacement therapy were left to the discretion of the physicians involved in care at that point in time, according to our usual ICU policies. Ventilation was initiated in pressure controlled mode and positive end expiratory pressure (PEEP) was applied, up to 20 cm of H_2_O. Patients who had persisting hypoxia were ventilated in the prone position and High Frequency Oscillation (HFO) was used in refractory cases. Bedside percutaneous tracheostomy was done in patients who required long term ventilation once their FiO_2_ requirements and PEEP levels allowed it to be carried out safely. The decision to wean and extubate were individualized, based on the discretion of the physicians attending the case. Patients were discharged to the wards once they had recovered sufficiently and their oxygen requirements had come down. The decision to discharge the patient home was made at the discretion of the ward physician. Data collected included epidemiological characteristics, the APACHE II score, blood investigations, ventilation and hemodynamic parameters, length of stay in the ICU and in the hospital as well as the final outcome. All patients except one were followed up until death or discharge from the hospital. The last patient is alive and in the hospital at the time of writing.

### Statistical analysis

Frequency analysis was carried out and expressed as mean ± standard deviation (SD) or median with 25^th^ to 75^th^ interquartile range (IQR) for continuous variables or number and percentage for categorical variables. Survivor and non-survivor characteristics were compared using Mann –Whitney test for non parametric variables. A two-tailed probability of *P* < 0.05 was considered significant. Risk of death analysis was done using a univariate Cox proportional hazards model, and hazard ratios calculated. Statistical analysis was done using Medcalc statistical software, version 11.1.1.

## Results

During a three month period between August to October 2009, 66 patients who presented with a flu-like illness and were confirmed positive for H1N1 2009 infection were admitted to our hospital. Of these, 38 patients required intensive care; seven of them were children, who were cared for in the pediatric ICU of our hospital. Thirty one adult patients were treated in the MICU during this period and represent the subjects of this study. By mid October, the number of new cases had diminished considerably. All except one of these patients have been discharged or died at the time of writing. There are three patients who are currently in our MICU who presented to us later, and are not part of this study.

The median age of our group of patients was 35 years with an interquartile range (IQR) of 28.2-42.8 [Table 1]. Maximum (11, 35.5%) patients were in the 30-40 age group. The youngest patient was 19 and the oldest, 55 years old. There were more male patients (58%) compared to females. Most of our patients presented with respiratory failure as single organ failure, reflected in the relatively low mean APACHE II score of 13.9. Nine patients (29%) had leukopenia, while seven (22.6%) had leukocytosis at presentation. Nine patients (29%) had a serum creatinine level of more than 1.2, while 11 (35.5%) patients had a platelet count of less than 150,000/mm^3^ at presentation.

**Table 1:** Patient characteristics, laboratory parameters

Patient characteristic	Value
Age (Median, IQR)	35 (28.2-42.8)
Sex	M = 18, F = 13
Onset to ICU, median, (IQR)	6 (5-7)
APACHE II score, mean, (SD)	13.9 (±6.9)
Haemoglobin (gm/dl), mean (SD),	13.5 (±2.2)
WBC count less than 4000/mm^3^ (No, %)	9 (29)
WBC count more than 11000/mm^3^ (No,%)	7 (22.6)
S. creatinine mg/dl, median (IQR)	1 (0.7-1.3)
AST (U/L), median (IQR)	66.5 (45.5-100.8)
ALT (U/L), median (IQR)	56 (42.5-79.5)
Platelet count (per mm^3^), median (IQR)	158,000 (113,000-189,000)

IQR: Interquartile range; SD: Standard deviation; AST: Aspartate aminotransferase, ALT: Alanine aminotransferase

Twenty patients (64.5%) had at least one risk factor [Table 2]. Obesity was the commonest risk factor, followed by hypertension. Three of our patients were pregnant, of whom two underwent invasive ventilation, while the third could be managed with non-invasive ventilation (NIV). All three were discharged home, one of them after 26 days of mechanical ventilation and 47 days in hospital. A 21-year-old patient presented as acute severe asthma and had to be intubated and ventilated; however, he improved rapidly and was extubated after 24 hours. There was one patient who was on immunosuppressive therapy after renal transplantation; however, he improved rapidly and did not require mechanical ventilation. One of our patients had antiphospholipid antibody syndrome and had presented to us with acute massive pulmonary embolism a year ago; he had undergone a pulmonary embolectomy and was on oral anticoagulants. Acute exacerbation of chronic obstructive pulmonary disease was the presenting feature in another case.

**Table 2:** Risk factors

Patient characteristic	Number (%)
Obesity (BMI > 30)	9 (29)
Hypertension	4 (12.9)
Pregnant	3 (9.7)
Diabetes	3 (9.7)
Asthma	2 (6.5)
Pre-existing renal failure	1 (3.2)
Post renal transplant, on immunosuppression	1 (3.2)
Chronic obstructive pulmonary disease	1 (3.2)

BMI: Body mass index = Weight in cms / Height in metres^2^

Twenty six (83.9%) patients required mechanical ventilation; four of them could be supported with NIV alone. Ventilation characteristics are represented in Table 3. NIV was tried initially on eight patients, out of which four required to be intubated and ventilated subsequently. Eighteen out of the 22 intubated patients (81.8%) required ventilator support for a week or more. Fifteen invasively ventilated patients (68.2%) required a PEEP of 15 cm of H_2_O or more. Nine patients (40.9%) were prone ventilated; each prone session lasted for a mean of 22.3 (2.5) hours. HFOV was employed in four patients as a rescue intervention for refractory hypoxia. A tracheostomy was done in six patients; this was done at a median of 14 days after intubation and ranged from 10 to 23 days. The mean tidal volume delivered was 402 ml and this was achieved at a median plateau pressure of 30 cm of H_2_O. Five patients developed a pneumothorax during mechanical ventilation. Two patients developed pneumothorax while on HFOV. Initial attempt at extubation failed in three patients. Out of this, one was extubated successfully after another 48 hours of ventilatory support; another required a tracheostomy; the third patient who failed extubation did not survive.

**Table 3:** Ventilation characteristics

Characteristic	Number
Non-invasive ventilation only (No, %)	4 (12.9)
Invasive ventilation (No, %)	22 (71)
No ventilator support (No, %)	5 (16.1)
No. of patients proned (No, %)	9 (40.9) of invasively ventilated patients
No. of prone sessions per patient, mean (SD)	3.1 (±0.6)
Max PEEP 10-15 cm of H_2_O (No, %)	9 (40.9)
Max PEEP 16-20 cm of H_2_O (No, %)	13 (59.1)
HFOV use (No, %)	4 (18.2)
Worst compliance, ml/cm of H_2_O, Median, (IQR)	22 (12-29)
Tidal volume, ml, mean (SD)	402.4 (±199.4)
Pplat, cm of H_2_O, median (IQR)	30 (26-38)
Tracheostomy	6 (27.3) of invasively ventilated patients

SD: Standard deviation; PEEP: Positive end-expiratory pressure; HFOV: High frequency oscillation ventilation; P_plat_: Plateau pressure

Acute Kidney Injury (AKI) as defined by the Acute Dialysis Quality Initiative group (ADQI)[6] was present on initial presentation in 6 (3.2%) patients at presentation. Overall, 11 (35.5%) patients suffered AKI at some stage during the course of their illness; four of them survived. Six patients with AKI required renal replacement therapy (RRT). Slow Low Efficiency Daily Dialysis (SLEDD) for 6-8 hours at a time was employed in all patients during the acute phase of illness; this was followed up with routine hemodialysis for four hours as per requirement once their condition stabilized. Out of six patients with AKI who required RRT, four survived. All survivors were off RRT at the time of discharge from hospital. Circulatory failure that required vasopressor support was encountered in 18 (58.1%) patients during the course of their illness; two of them had evidence of significant global left ventricular dysfunction on the echocardiogram. This was transient and reversed completely in both patients within a week’s time. Of the patients who developed circulatory failure, six (33.3%) died. Severe hypotension refractory to fluid resuscitation and vasopressor support along with refractory hypoxia was seen in one patient at the time of presentation. This patient progressively worsened and died within 24 hours of admission to our unit. Refractory seizures were seen in one patient that required multiple anti-seizure medications for control. This patient continues to be in altered sensorium and is being cared for in the ward at the time of writing.

One patient, a 19-year-old boy, who was the youngest in our series, developed severe critical illness related polyneuropathy and required several weeks of rehabilitation; he improved subsequently and has minimal residual disability at the time of writing.

Of the 31 patients admitted to the MICU during the study period, 25 (80.6%) were alive at 28 days; of these, all except one were discharged subsequently. One patient, who presented with an encephalitis- like picture had refractory seizures which required multiple anti-seizure medications for control. At the time of writing, 44 days after admission to hospital, he is still in the ward with significant neurological deficits. Comparisons of survivor and non-survivor characteristics are depicted in Table 4. The median duration of ventilator support in all ventilated patients was 10 days with an IQR of 4-22 days. The median duration of ICU stay was 10 days with an IQR of 5-17 days. Patients stayed in hospital for a median of 15 days with an IQR of 8-22.5 days. The shortest hospital stay was 2 days and the longest, 58 days. On a univariate Cox proportional hazards model, increasing APACHE II scores were associated with an increased risk of death (Hazard Ratio: 1.1; CI: 1.08 -1.2; *P* = 0.04). Of all ventilated patients, the median tidal volumes used on day 1 were lower at 350 ml (IQR: 320-388) in non-survivors versus 500 ml (IQR: 450-550) in survivors, which was statistically significant.(CI: 30 -200; *P* = 0.01)

**Table 4:** Comparison - survivors vs. non-survivors

Patient characteristics	Survivors	Non-survivors	Hazard ratio (95% CI)	P value
Age, median (IQR)	35 (28–45)	35.5 (31.3–37.5)	0.97 (0.89-1.1)	0.57
Male (No, %)	16 (84.2)	3(15.8)		
Female (no, %)	9 (75)	3 (25)	2.8 (0.51- 15.12)	0.24
Onset to ICU, median (IQR)	6 (5-7)	6.5 (5.2-7)	1.01 (0.76-1.35)	0.92
APACHE II (Median, IQR)	13 (8-16)	17.5 (14.8-22.5)	1.2 (1.11-1.22)	0.04
Obese (No, %)	5 (55.6)	4 (44.4)	4.89 (0.9-26.7)	0.06
Creatinine, mg/dl day1(median, IQR)	1 (0.7-1.3)	1 (0.8-1.9)	1.12 (0.5-2.51)	0.77
Av. Vt day 1, ml, median, (IQR)	500 (450-550)	350 (350-388)		0.01
Av. Pplat, cm of H_2_O day 1 median (IQR)	29.5 (26-35.3)	36.5 (30.7-40.7)		0.17
Worst compliance, ml/ cm of H_2_O, median (IQR)	21 (19.5-29.3)	12 (12-14)		0.02
Worst P/F, median (IQR)	86.5 (62.2-112.7)	65 (65-80)		0.12
Highest PEEP, cm of H_2_O, median (IQR)	15 (10-20)	20 (18-20)		0.14
AKI (No, %)	7/25 (28)	4/6 (66.7)	3.64 (0.67-19.8)	0.14

CI: Confidence interval; IQR: Interquartile range; Vt: Tidal volume; P_plat_: Plateau pressure; P/F: PaO_2_/FiO_2_ ratio; PEEP: Positive end expiratory pressure; AKI: Acute kidney injury

## Discussion

Concerns have been raised regarding the possibility of a pandemic flu outbreak in India – especially in the light of the previous pandemics of 1917, 1957 and 1968 that had a significant impact in this country. The level of preparedness to cope with a rapidly spreading illness with a potential to affect several thousands of people within a short time has been debated, especially given the limited facilities available in the Indian public health system. Although the current outbreak seems to have touched a nadir in India as well as the rest of the world, the lessons learnt could be used to plan more efficiently to handle epidemics of such magnitude in the future. At the time of writing, the city of Bangalore has recorded the third largest number of cases, after Delhi and Pune.[7] It is against this background that we present our series of H1N1 patients from the ICU of a corporate, tertiary care hospital.

Most our patients were young and without significant co-morbidities in keeping with world- wide experience;[8–13] more than 90% of them were less than 50 years of age. This is in contrast with the pattern seen in seasonal influenza, where relatively more patients in the extremes of age group are affected.[14] The preponderance of the 2009 H1N1 virus for the younger age group may be due to the immunity present in the older age groups through cross reactive antibodies. In a study by Hancock *et al*, 34% of subjects more than 50 years, born before 1950 were shown to have such antibodies compared to only 4% in those born after 1980.[15]

The median duration between onset of symptoms and admission to our unit was six days in our series; this did not significantly differ between survivors and non-survivors. However, 17 (54.8%) of our patients were transferred to our unit from other hospitals within the city and five of them were already intubated and ventilated prior to transfer.

The mean APACHE II score in our series was relatively low at 13.9 (±6.9); this was largely because most of our patients presented with ARDS and respiratory failure as a single organ failure with significant hypoxemia and a wide alveolar-arterial oxygen tension gradient. The median APACHE II scores were significantly higher in non-survivors compared to survivors. APACHE II scores were also significantly higher in non-survivors at 28 days in a Canadian study.[9] Many of our patients were hypotensive and required fluid resuscitation and vasopressor support in the first few days. We feel that the use of heavy sedation initially could have contributed significantly to the low blood pressures observed.

We observed AKI in 35.5% of our patients; 4 out of the 6 patients who died had evidence of AKI. The incidence of AKI was 7.1% in the Canadian series[9] while a case series from Mexico reported six patients with acute renal failure, of whom five had died.[12]

Obesity was the commonest risk factor present in our series. The increased propensity for H1N1 2009 infection to affect obese subjects has been documented in other reports;[9,10] however, it is not clear as to why obesity adds to the risk of H1N1 infection. Unlike 2009 H1N1 infection, obesity has not been identified as a risk factor for seasonal influenza. It is also interesting to note that obesity has not been linked to reduced survival associated with 2009 H1N1 infection in the two large studies on intensive care patients published so far. Some studies suggest increased mortality and morbidity in obese critically ill patients;[16–18] on the other hand, it is possible that obesity may even have a protective effect in the ICU population.[19,20] Three of our patients were pregnant; one of them was admitted at 14 weeks of gestation and remained severely hypoxic for several weeks, before she began to improve. She required ventilator support for 26 days and remained in hospital for 47 days. She was symptom free and oxygenating normally at the time of discharge from the hospital and the fetus appears to have suffered no damage. The admission rate for H1N1 infection in pregnant women was more than that in the general population in a study of 34 pregnant women reported to the CDC during the first month of the outbreak in the US. Pregnant women might also be at increased risk of complications from H1N1 infection.[21] In the ANZICS study, 9.1% of patients admitted to intensive care were pregnant although pregnant women represented only 1% of the general population in Australia and New Zealand.[8]

Severe hypoxia was the predominant feature in all our patients. All of them had a worst PaO_2_ / FiO_2_ (P/F) ratio of less than 200. In 12 (38.7%) patients, the P/F ratio was less than 100; it was less than 150 in 27 (87.1%) patients. Relatively high levels of PEEP were employed in all ventilated patients; nearly 60% of all ventilated patients required a PEEP of between 15 and 20 cm of H_2_O. The PEEP levels we used seem to be higher compared to other studies.[9,13] We employed higher levels of PEEP in non-survivors, but this was not statistically significant.

In our study, non-survivors had stiffer lungs; the worst compliance recorded was significantly lower in those who died. This was reflected in the tidal volumes measured as well, which were significantly lower in those who died. We resorted to prone ventilation in patients who remained hypoxic after optimal levels of PEEP; HFOV was used in refractory cases. We did not use nitric oxide or extracorporeal membrane oxygenation extracorporeal membrane oxygenation (ECMO) in any patient.

The reason for the severe hypoxemia seen in H1N1 patients is being debated. It is hypothesized that secondary immune responses consequent upon high rates of viral replication and the ensuing cytokine storm may be responsible for severe pulmonary damage, although no data exists to substantiate this. Five out of ten patients had evidence of pulmonary embolism on computerized tomography (CT) in a series of 10 patients admitted to a surgical ICU. Two patients had a hypercoagulable state and bilateral iliofemoral deep vein thrombosis was observed in one.[22] Agarwal *et al*. observed pulmonary emboli in 5 out of 14 H1N1 patients who were mechanically ventilated.[23] It is possible that pulmonary emboli may account for the severe hypoxemia that is often seen in some of these patients.

We diagnosed H1N1 infection by RT-PCR testing on suspected cases at the time of initial presentation. False negative RT-PCR has been observed in several cases. Initial testing was negative in five out of 32 patients in one study;[11] repeat testing confirmed infection in these patients. In the ANZICS series, five out of 722 patients who were negative on RT-PCR testing were confirmed on serological analysis.[8] Indeed, we observed several patients during the study period with clinical and radiological features suggestive of H1N1 infection but tested negative; it is possible that RT-PCR may have been falsely negative for 2009 H1N1 infection in some of these patients.

Our report is the first on 2009 H1N1 infection from an Indian ICU setting. Although the study sample is small and from a single centre, we were able to follow up all patients, except one, till death or discharge from hospital. Commensurate with world wide experience, most of our patients were otherwise fit and well, young adults who presented with breathing difficulty and severe hypoxemia. More than half of all patients (including children) who presented to our tertiary care hospital required mechanical ventilation, many of them for two weeks or more. We found that with appropriate ventilator support and good quality intensive care, most of our patients improved over time and could be discharged home. With limited facilities available in the public sector, the cost implications of this type of care may be huge. We observed that many of our patients, especially those who died, had very stiff lungs that became hard to ventilate. The use of ECMO has been shown to be of benefit in this situation;[24] in the event of a future outbreak, planning should focus on transferring patients with refractory hypoxemia to units that have the facility to do this. The mechanism of causation of severe hypoxia in H1N1 infection needs further research; in addition to extensive alveolar consolidation, pulmonary embolism is a possibility. Pooling of data from centers across the country would certainly help get a better understanding of similar outbreaks and help administrators plan for such disasters in the future.

## Conclusions

2009 H1N1 infection resulted in respiratory failure requiring prolonged mechanical ventilation in relatively young patients. Obesity was the most common risk factor. Extra-pulmonary involvement included circulatory and renal failure. Non-survivors had higher APACHE II scores and relatively non-compliant lungs.
